# Establishment and validation of a nomogram to predict postoperative anemia after total hip arthroplasty

**DOI:** 10.1186/s12891-024-07264-w

**Published:** 2024-02-14

**Authors:** Xiang Li, Hong-yang Jiang, Yong-jie Zhao, Si-zhuo Liu, Ling-xiao Pan

**Affiliations:** 1 Department of Orthopedics and Sports Medicine, Li Huili Hospital Affiliated to Ningbo University, 1111 Jiangnan Street, Ningbo, 315000 China; 2 Health Science Center, Ningbo University, 818 Fenghua Street, Ningbo, 315211 China

**Keywords:** Total hip replacement, Risk factor, Anemia, Nomogram, Serum amyloid A

## Abstract

**Background:**

Anemia is a common complication of total hip arthroplasty (THA). In this study, we evaluated the preoperative risk factors for postoperative anemia after THA and developed a nomogram model based on related preoperative and intraoperative factors.

**Methods:**

From January 2020 to May 2023, 927 THA patients at the same medical center were randomly assigned to either the training or validation cohort. The correlation between preoperative and intraoperative risk factors and postoperative anemia after THA was evaluated using univariate and multivariate logistic regression analysis. A nomogram was developed using these predictive variables. The effectiveness and validation for the clinical application of this nomogram were evaluated using the concordance index (C-index), receiver operating characteristic (ROC) curve, calibration curve, and decision curve analysis (DCA).

**Results:**

Through univariate and multivariate logistic regression analysis, 7 independent predictive factors were identified in the training cohort: Lower body mass index (BMI), extended operation time, greater intraoperative bleeding, lower preoperative hemoglobin level, abnormally high preoperative serum amyloid A (SAA) level, history of cerebrovascular disease, and history of osteoporosis. The C-index of the model was 0.871, while the AUC indices for the training and validation cohorts were 84.4% and 87.1%, respectively. In addition, the calibration curves of both cohorts showed excellent consistency between the observed and predicted probabilities. The DCA curves of the training and validation cohorts were high, indicating the high clinical applicability of the model.

**Conclusions:**

Lower BMI, extended operation time, increased intraoperative bleeding, reduced preoperative hemoglobin level, elevated preoperative SAA level, history of cerebrovascular disease, and history of osteoporosis were seven independent preoperative risk factors associated with postoperative anemia after THA. The nomogram developed could aid in predicting postoperative anemia, facilitating advanced preparation, and enhancing blood management. Furthermore, the nomogram could assist clinicians in identifying patients most at risk for postoperative anemia.

## Background

With nearly a century of development and significant advances in implant materials, surgical techniques, and complication prevention, total hip arthroplasty (THA) has become the standard surgical treatment for end-stage hip diseases, such as femoral neck fractures, osteonecrosis of the femoral head, and hip osteoarthritis. Known for its efficacy in alleviating pain, restoring joint function, and significantly enhancing the quality of life in patients, THA has earned the accolade of the "Surgery of the Century" for the twentieth century [1]. Currently, over a million THA procedures are performed annually worldwide [2]. However, despite employing various measures, such as proper exposure of the surgical field and the use of intravenous tranexamic acid (TXA), anemia remains a frequent complication of THA, especially in older patients with serious complications [3]. Related literature suggests that this could be due to several factors, such as sex, age, American Society of Anesthesiologists (ASA) score, and complications [4, 5].

Recognized as a risk factor, postoperative anemia contributes to a decline in patient quality of life (QOL) and deterioration in the prognosis of patients, even when the condition does not necessitate a blood transfusion [6]. Anemia can lead to a range of adverse outcomes, including heart failure [7], weakness [8], and even death [9]. Perioperative blood management is essential in preventing these complications. Gilbody et al. reported the incidence of postoperative blood transfusion after THA to be between 16.9% and 50%, with about half of postoperative blood transfusions exceeding 2 units [3, 10]. However, transfusion can lead to transfusion-related complications, increase the risk of bacterial infection and death, and may also increase economic burden [4, 11]. Therefore, it is critically important to identify patients with potential anemia risk. This can help clinicians prevent postoperative anemia through various interventions during the perioperative period.

We aimed to investigate the risk factors of postoperative anemia following THA, develop a nomogram of anemia risk, and assess the accuracy and clinical applicability of the nomogram.

## Methods

This study retrospectively analyzed 927 patients who underwent THA at our hospital from January 2020 to May 2023.

The inclusion criteria were as follows: (1) a posterior-lateral surgical approach; (2) meeting the indications for THA with a first-time unilateral THA procedure scheduled; (3) accurate recording of the medical history and complete data in electronic medical records; and (4) no iron supplementation or blood transfusion before admission.

The exclusion criteria were as follows: (1) revision hip arthroplasty; (2) coagulation or hematopoietic function disorders; (3) history of using antiplatelet drugs, nonsteroidal anti-inflammatory drugs, or vasoactive drugs within one week preceding the surgery; (4) ASA score above grade III; and (5) patients with bone tumors or other malignant tumors.

All surgeries were performed by senior chief physicians, and the same intraoperative blood management measures were implemented. The included patients were randomly assigned into a training cohort and a validation cohort.

### Data collection

In this study, 27 variables were utilized for modeling and analysis which were collected via the electronic medical record system. Preoperative data included age, sex, body mass index (BMI), the reason for surgery (femoral neck fracture [FNF], osteonecrosis of the femoral head [ONFH], osteoarthritis of hip [OAH]), intraoperative blood loss, operation time, type of anesthesia (general anesthesia [GA], combined spinal epidural anesthesia [CSEA]), smoking history, drinking history, history of hypertension, history of diabetes, history of cardiovascular disease, history of cerebrovascular disease, and history of osteoporosis. Lab data included total protein (TP), albumin (ALB), globulin (GLB), serum amyloid A (SAA), serum iron (IRON), total iron binding capacity (TIBC), C-reactive protein (CRP), red blood cell (RBC) count, hemoglobin (HB), hematocrit (HCT), mean corpuscular volume (MCV), mean corpuscular hemoglobin (MCH), and mean corpuscular hemoglobin concentration (MCHC).

Postoperative anemia was defined as Hb < 9 g/dL on postoperative day 3 according to the literature [12].

### Statistical analysis

Statistical analysis was performed using SPSS version 22 (IBM Corp., Armonk, NY). Continuous variables are presented as mean ± standard deviation and categorical variables as counts (%). Continuous variables were compared using Student's t-test or the Mann–Whitney U test, and categorical variables using the Chi-square test or Fisher's exact test. Subsequently, univariate and multivariate logistic regression analyses were conducted on all variables in the training and validation cohorts to exclude irrelevant risk factors. Independent risk factors obtained were utilized to construct a nomogram model with the rms package of R software (version 4.1.0). Finally, the concordance index (C-index), the area under the receiver operating characteristic (ROC) curve (AUC), the calibration curve, and the Decision Curve Analysis (DCA) were used to evaluate the predictive ability and performance of the risk model. The C-index assesses the prediction accuracy and discrimination ability of the nomogram. The AUC evaluates the predictive ability of the model. The calibration curve was used to compare the actual and predicted risks. The clinical utility of the nomogram was evaluated by DCA based on net benefit and threshold probability. A *p* < 0.05 was used as the level of significance in statistical tests.

## Results

We assessed 1,025 patients who underwent THA at the Li Huili Hospital of the Ningbo Medical Center from January 2020 to May 2023. Of these, 927 patients met the inclusion criteria. The patients were randomly assigned with 616 individuals in the training cohort and 311 in the validation cohort. The baseline characteristics of the patients in both cohorts, including demographic characteristics, intraoperative data, and lab data, were largely similar. The relevant variables and detailed information are shown in Table 1.

**Table 1:** Basic characteristics of patients from both training and validation cohort

*Variables*	*Training cohort (N* = *616)*	*Validation cohort (N* = *311)*	*p-value*
*Age (years)*	*70.50* ± *13.10*	*69.76* ± *13.48*	*0.490*
*BMI (kg/m*^*2*^*)*	*22.94* ± *3.21*	*22.90* ± *2.98*	*0.888*
*Sex*			*0.594*
*Male*	*126 (20.5%)*	*59 (19.0%)*	
*Female*	*490 (79.5%)*	*252 (81.0%)*	
*Reasons of operation*			*0.450*
*FNF*	*197 (32.0%)*	*97 (31.2%)*	
*ONFN*	*257 (41.7%)*	*142 (45.7%)*	
*OAH*	*162 (26.3%)*	*72 (23.2%)*	
*Operation time (min)*	*91.62* ± *30.21*	*92.55* ± *31.68*	*0.977*
*Intraoperative blood loss (mL)*	*118.84* ± *112.39*	*132.19* ± *126.91*	*0.444*
*Type of anesthesia*			*0.648*
*GA*	*321 (52.1%)*	*167 (53.7%)*	
*CSEA*	*295 (47.9%)*	*144 (46.3%)*	
*Smoking*			*0.347*
*No*	*529 (85.9%)*	*274 (88.1%)*	
*Yes*	*87 (14.1%)*	*37 (11.9%)*	
*Drinking*			*0.334*
*No*	*535 (86.9%)*	*277 (89.1%)*	
*Yes*	*81 (13.1%)*	*34 (10.9%)*	
*Hypertension*			*0.395*
*No*	*275 (44.6%)*	*148 (47.6%*	
*Yes*	*341 (55.4%)*	*163 (52.4%*	
*Diabetes*			*0.199*
*No*	*485 (78.7%)*	*256 (82.3)*	
*Yes*	*131 (21.3)*	*55 (17.7%)*	
*Cardiovascular disease*			*0.783*
*No*	*541 (87.8%)*	*278 (89.4%)*	
*Yes*	*75 (12.2%)*	*33 (10.6%)*	
*Cerebrovascular disease*			*0.276*
*No*	*516 (83.8%)*	*269 (86.5%)*	
*Yes*	*100 (16.2%)*	*42 (13.5%)*	
*Osteoporosis*			*0.517*
*No*	*378 (61.4%)*	*184 (59.2%)*	
*Yes*	*238 (38.6%)*	*127 (40.8%)*	
*TP (g/L)*	*65.70* ± *7.40*	*66.41* ± *7.19*	*0.164*
*ALB(g/L)*	*37.76* ± *4.99*	*38.19* ± *4.76*	*0.208*
*GLB (g/L)*	*27.82* ± *5.10*	*28.20* ± *5.33*	*0.330*
*SAA (mg/L)*	*81.70* ± *94.98*	*93.45* ± *108.59*	*0.056*
*IRON (μmol/L)*	*13.39* ± *12.30*	*13.10* ± *13.75*	*0.627*
*TIBC (μmol/L)*	*46.97* ± *9.33*	*47.20* ± *10.28*	*0.787*
*CRP (mg/L)*	*23.44* ± *32.77*	*27.54* ± *36.60*	*0.183*
*RBC (1,012/L)*	*3.94* ± *1.58*	*3.94* ± *0.67*	*0.450*
*Hb(g/L)*	*120.01* ± *20.13*	*121.47* ± *27.32*	*0.901*
*HCT (%)*	*36.04* ± *6.36*	*36.42* ± *5.85*	*0.395*
*MCV (fL)*	*93.30* ± *6.20*	*93.06* ± *6.81*	*0.823*
*MCH (pg)*	*31.11* ± *2.42*	*30.86* ± *2.78*	*0.519*
*MCHC (g/L)*	*333.08* ± *9.83*	*330.82* ± *11.68*	*0.055*
*Anemia*			*0.376*
*No*	*381 (61.9%)*	*183 (58.8%)*	
*Yes*	*235 (38.1%)*	*128 (41.2%)*	

*BMI* body mass index, *FNF* femoral neck fracture, *ONFN* osteonecrosis of the femoral head, *OAH* hip osteoarthritis, *GA* General Anesthesia, *CSEA* Combined Spinal Epidural Anesthesia, *TP* Total Protein, *ALB* albumin, *GLB* globulin, *SAA* serum amyloid-a, *IRON* serum iron, *TIBC* Total Iron binding capacity, *CRP* C-reactive protein, *RBC* red blood cell, *Hb* Hemoglobin, *HCT* Hematocrit, *MCV* mean corpuscular volume, *MCH* mean corpuscular hemoglobin, *MCHC* mean corpuscular hemoglobin concentration

Data are presented as n (%) or mean ± standard deviation

Bold represents *p*-value < 0.05

### Potential risk factors for postoperative anemia

Within 3 days following THA, 235 patients from the training cohort developed anemia, representing 38.1% of the cohort. Univariate logistic regression analysis revealed that age, sex, BMI, operation time, intraoperative blood loss, history of hypertension, history of diabetes, history of cerebrovascular disease, history of osteoporosis, TP, ALB, SAA, RBC, HB, HCT, and MCHC were associated with postoperative anemia in this cohort. Multivariate logistic regression was subsequently employed to further analyze these variables, leading to the identification of independent risk factors, which included BMI, operation time, intraoperative blood loss, history of cerebrovascular disease, history of osteoporosis, SAA, and HB, as shown in Table 2.

**Table 2:** Postoperative anemia of THA: Univariate and multivariate analysis of possible risk factors in the training cohort

*Variables*	*Univariate analysis OR (95% CI)*	*p-value*	*Multivariate analysis Adjusted OR (95% CI)*	*p-value*
*Age (years)*	*1.026 (1.012–1.039)*	< *0.001*	*0.984 (0.964–1.005)*	*0.127*
*BMI (kg/m*^*2*^*)*	*0.861 (0.812–0.913)*	< *0.001*	*0.903 (0.840–0.970)*	*0.005*
*Sex*				
*Male*	*1*		*1*	
*Female*	*1.631 (1.068–2.492)*	*0.024*	*0.878 (0.482–1.600)*	*0.671*
*Reasons of operation*				
*FNF*	*1*			
*ONFN*	*1.420 (0.967–2.084)*	*0.074*		
*OAH*	*1.030 (0.666–1.593)*	*0.895*		
*Operation time (min)*	*1.011 (1.005–1.016)*	< *0.001*	*1.011 (1.003–1.018)*	*0.006*
*Intraoperative blood loss (mL)*	*1.002 (1.001–1.004)*	*0.002*	*1.003 (1.000–1.005)*	*0.017*
*Type of anesthesia*				
*GA*	*1*			
*CSEA*	*1.291 (0.937–1.798)*	*0.117*		
*Smoking*				
*No*	*1*			
*Yes*	*1.458 (0.923–2.303)*	*0.106*		
*Drinking*				
*No*	*1*			
*Yes*	*1.202 (0.748–1.932)*	*0.447*		
*Hypertension*				
*No*	*1*		*1*	
*Yes*	*1.915 (1.370–2.678)*	< *0.001*	*1.400 (0.873–2.245)*	*0.163*
*Diabetes*				
*No*	*1*		*1*	
*Yes*	*1.497 (1.013–2.212)*	*0.043*	*1.371 (0.801–2.347)*	*0.249*
*Cardiovascular disease*				
*No*	*1*			
*Yes*	*1.239 (0.759–2.022)*	*0.391*		
*Cerebrovascular disease*				
*No*	*1*		*1*	
*Yes*	*3.780 (2.410–5.928)*	< *0.001*	*2.485 (1.369–4.514)*	*0.003*
*Osteoporosis*				
*No*	*1*		*1*	
*Yes*	*3.13 (2.227–4.398)*	< *0.001*	*2.273 (1.464–3.529)*	< *0.001*
*TP (g/L)*	*0.958 (0.936–0.980)*	< *0.001*	*1.004 (0.960–1.050)*	*0.868*
*ALB (g/L)*	*0.930 (0.899–0.963)*	< *0.001*	*1.118 (1.038–1.205)*	*0.003*
*GLB (g/L)*	*0.982 (0.951–1.014)*	*0.268*		
*SAA (mg/L)*	*1.004 (1.002–1.006)*	< *0.001*	*1.003 (1.001–1.005)*	*0.014*
*IRON (μmol/L)*	*1.005 (0.991–1.018)*	*0.499*		
*TIBC (μmol/L)*	*0.972 (0.954–0.990)*	*0.002*	*0.991 (0.966–1.018)*	*0.517*
*CRP (mg/L)*	*1.003 (0.999–1.008)*	*0.162*		
*RBC (1,012/L)*	*0.208 (0.152–0.286)*	< *0.001*	*0.842 (0.386–1.839)*	*0.666*
*HB (g/L)*	*0.935 (0.924–0.947)*	< *0.001*	*0.936 (0.894–0.980)*	*0.005*
*HCT (%)*	*0.830 (0.800–0.861)*	< *0.001*	*0.972 (0.860–1.099)*	*0.952*
*MCV (fL)*	*1.010 (0.984–1.037)*	*0.436*		
*MCH (pg)*	*0.988 (0.923–1.057)*	*0.724*		
*MCHC (g/L)*	*0.971 (0.954–0.988)*	*0.001*	*0.979 (0.951–1.009)*	*0.164*

*OR* odds ratio, *CI* confidence interval, *BMI* body mass index, *FNF* femoral neck fracture, *ONFN* osteonecrosis of the femoral head, *OAH* hip osteoarthritis, *GA* General Anesthesia, *CSEA* Combined Spinal Epidural Anesthesia, *TP* Total Protein, *ALB* albumin, *GLB* globulin, *SAA* serum amyloid-a, *IRON* serum iron, *TIBC* Total Iron binding capacity, *CRP* C-reactive protein, *RBC* red blood cell, *HB* Hemoglobin, *HCT* Hematocrit, *MCV* mean corpuscular volume, *MCH* mean corpuscular hemoglobin, *MCHC* mean corpuscular hemoglobin concentration

Data are presented as n (%) or mean ± standard deviation

Bold represents *p*-value of the variable that is less than 0.05

In terms of BMI, lower values were observed to be more likely to result in postoperative anemia (adjusted odds ratio [OR]: 0.903, 95% confidence interval [CI], 0.840–0.970, *p* = 0.005). Longer operation times and larger volumes of intraoperative blood loss also increased the likelihood of postoperative anemia (adjusted OR: 1.011, 95% CI, 1.003–1.018, *p* = 0.006; adjusted OR: 1.003, 95% CI, 1.000–1.005, *p* = 0.017; respectively). A history of cerebrovascular disease and osteoporosis were also predictive indicators of postoperative anemia (adjusted OR: 2.485, 95% CI, 1.369–4.514, *p* = 0.003; adjusted OR: 2.273, 95% CI, 1.464–3.529, *p* < 0.001; respectively). Lower preoperative HB could lead to postoperative anemia (adjusted OR: 0.936, 95% CI, 0.894–0.980, *p* = 0.005). Additionally, higher preoperative SAA was found to potentially lead to postoperative anemia (adjusted OR: 1.003, 95% CI, 1.001–1.005, *p* = 0.014).

As shown in Table 3, a similar analysis was performed in the validation cohort. Age, BMI, operation time, intraoperative blood loss, history of hypertension, history of stroke, history of osteoporosis, TP, ALB, SAA, RBC, Hb, HCT, and MCHC were found to be associated with postoperative anemia, while sex, diabetes, and TP did not show similar effects. Furthermore, CSEA and IRON emerged as risk factors. However, only BMI, operation time, intraoperative blood loss, history of osteoporosis, SAA, and Hb were ultimately identified as strong risk factors in the multivariate logistic regression analysis.

**Table 3:** Postoperative anemia of THA: univariate and multivariate analysis of possible risk factors in the validation cohort

*Variables*	*Univariate analysis OR (95% CI)*	*p-value*	*Multivariate analysis Adjusted OR (95% CI)*	*p-value*
*Age (years)*	*1.019 (1.002–1.037)*	*0.029*	*1.011 (0.979–1.043)*	*0.512*
*BMI (kg/m*^*2*^*)*	*0.878 (0.810–0.952)*	*0.002*	*0.899 (0.797–1.014)*	*0.082*
*Sex*				
*Male*	*1*			
*Female*	*0.833 (0.529–1.669)*	*0.833*		
*Reasons of operation*				
*FNF*	*1*			
*ONFN*	*0.731 (0.433–1.232)*	*0.239*		
*OAH*	*0.735 (0.396–1.366)*	*0.331*		
*Operation time (min)*	*1.024 (1.015–1.033)*	< *0.001*	*1.032 (1.018–1.046)*	< *0.001*
*Intraoperative blood loss (mL)*	*1.006 (1.004–1.009)*	< *0.001*	*1.004 (1.001–1.007)*	*0.014*
*Type of anesthesia*				
*GA*	*1*		*1*	
*CSEA*	*2.212 (1.396–3.505)*	*0.001*	*2.066 (1.018–4.194)*	*0.065*
*Smoking*				
*No*	*1*			
*Yes*	*1.598 (0.803–3.181)*	*0.182*		
*Drinking*				
*No*	*1*			
*Yes*	*1.495 (0.732–3.054)*	*0.269*		
*Hypertension*				
*No*	*1*		*1*	
*Yes*	*2.008 (1.266–3.185)*	*0.003*	*1.203 (0.615–2.352)*	*0.589*
*Diabetes*				
*No*	*1*			
*Yes*	*1.480 (0.824–2.656)*	*0.189*		
*Cardiovascular disease*				
*No*	*1*			
*Yes*	*0.921 (0.440–1.927)*	*0.828*		
*Cerebrovascular disease*				
*No*	*1*		*1*	
*Yes*	*2.661 (1.362–5.198)*	*0.004*	*1.748 (0.615–2.352)*	*0.232*
*Osteoporosis*				
*No*	*1*		*1*	
*Yes*	*2.252 (1.415–3.582)*	*0.001*	*2.117 (1.112–4.030)*	*0.022*
*TP (g/L)*	*0.981 (0.951–1.013)*	*0.242*		
*ALB (g/L)*	*0.932 (0.887–0.979)*	*0.005*	*1.066 (0.983–1.156)*	*0.125*
*GLB (g/L)*	*1.022 (0.979–1.066)*	*0.325*		
*SAA (mg/L)*	*1.006 (1.004–1.009)*	< *0.001*	*1.004 (1.000–1.007)*	*0.044*
*IRON (μmol/L)*	*0.969 (0.942–0.997)*	*0.027*	*0.986 (0.953–1.021)*	*0.428*
*TIBC (μmol/L)*	*1.007 (0.985–1.029)*	*0.559*		
*CRP (mg/L)*	*1.003 (0.997–1.009)*	*0.317*		
*RBC (1,012/L)*	*0.251 (0.163–0.386)*	< *0.001*	*0.341 (0.098–1.190)*	*0.092*
*HB (g/L)*	*0.944 (0.929–0.959)*	< *0.001*	*0.980 (0.917–1.048)*	*0.006*
*HCT (%)*	*0.832 (0.789–0.877)*	< *0.001*	*0.947 (0.792–1.133)*	*0.553*
*MCV (fL)*	*0.989 (0.957–1.022)*	*0.512*		
*MCH (pg)*	*0.955 (0.881–1.036)*	*0.272*		
*MCHC (g/L)*	*0.973 (0.954–0.993)*	*0.007*	*0.954 (0.917–0.993)*	*0.051*

*OR* odds ratio, *CI* confidence interval, *BMI* body mass index, *FNF* femoral neck fracture, *ONFN* osteonecrosis of the femoral head, *OAH* hip osteoarthritis, *GA* General Anesthesia, *CSEA* Combined Spinal Epidural Anesthesia, *TP* Total Protein, *ALB* albumin, *GLB* globulin, *SAA* serum amyloid-a, *IRON* serum iron, *TIBC* Total Iron binding capacity, *CRP* C-reactive protein, *RBC* red blood cell, *HB* Hemoglobin, *HCT* Hematocrit, *MCV* mean corpuscular volume, *MCH* mean corpuscular hemoglobin, *MCHC* mean corpuscular hemoglobin concentration

Data are presented as n (%) or mean ± SD

Bold represents *p*-value of the variable that is less than 0.05

### Establishment and validation of the nomogram for predicting postoperative anemia

**Fig. 1:**
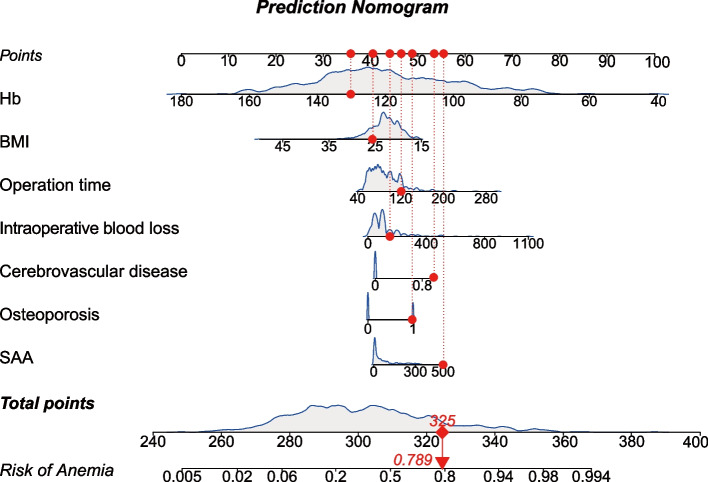
Nomogram for calculating the risk of postoperative anemia of patients who underwent THA. BMI, body mass index; HB, hemoglobin; SAA, serum amyloid A; THA, total hip arthroplasty

The application of the nomogram is simple, which involves summing the scores of each prognostic index to compute the total score and subsequently using this total score to calculate the probability of postoperative anemia. For example, according to this model as outlined in Fig. 1, a patient with a preoperative Hb of 130 g/L (35 points), SAA of 500 (55 points), BMI of 25 kg/m^2^ (40 points), history of cerebrovascular disease (54 points), and history of osteoporosis (49 points) undergoing THA surgery, with an operation time of 120 min (47 points) and intraoperative blood loss of 150 mL (45 points), accumulates a total score of 325 points. By referring to the total points axis, a value of 0.789 is obtained, thus predicting that the risk of developing postoperative anemia is roughly 78.9%.

**Fig. 2:**
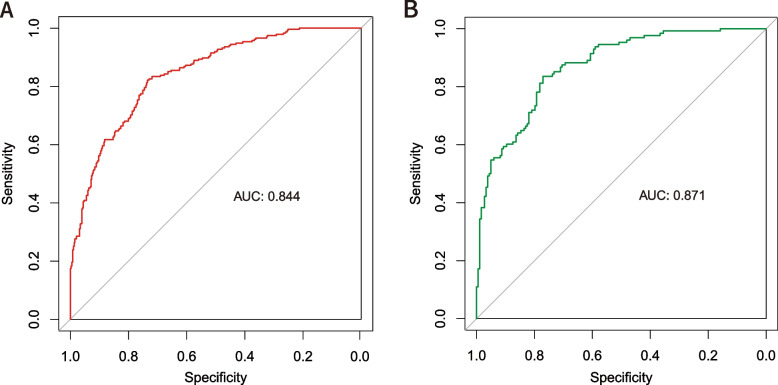
The receiver operating characteristic (ROC) curve of training cohort (**A**) validation cohort (**B**). AUC, area under the curve

**Fig. 3:**
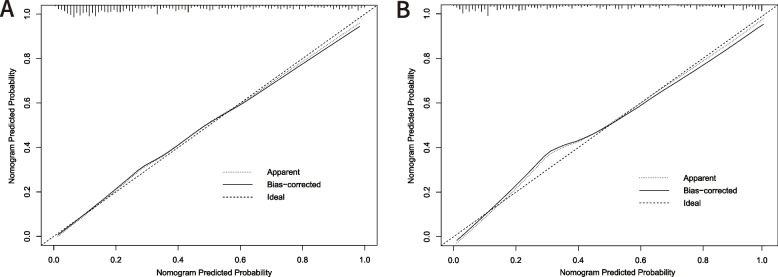
The calibration curve of the (**A**) training cohort and (**B**) validation cohort

**Fig. 4:**
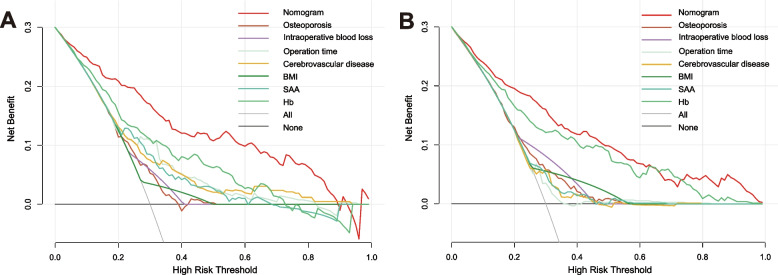
The decision curve analysis of the (**A**) training cohort and (**B**) validation cohort. BMI, body mass index; SAA, serum amyloid-a; Hb, hemoglobin

The accuracy of the nomogram model was evaluated via the validation of the training and validation cohorts. The C-index of this model is 0.871, indicating that this model possesses high predictive accuracy. An ROC curve was constructed and the AUCs of the training and validation cohorts were calculated. The AUC in the training cohort was 84.4%, and the AUC in the validation cohort was 87.1% (Fig. 2A, B), suggesting the strong predictive power of this model. The calibration curve demonstrates excellent consistency between the observed and predicted probabilities of the model (Fig. 3A, B). The DCA reveals that this nomogram model can serve as a reliable predictive tool for postoperative anemia following THA surgery (Fig. 4A, B).

## Discussion

As THA techniques mature and socioeconomic conditions improve, the number of patients undergoing THA continues to increase. However, postoperative anemia following THA remains an issue. Shander et al. underscored that such anemia signals treatment failure [12]. THA patients are typically older and frequently suffer from various comorbidities, such as cardiovascular disease, and the negative impact of anemia in this population has been well-documented, even for mild or moderate cases [6]. Severe postoperative anemia often necessitates a transfusion, which not only increases economic costs but also introduces risks, including heart failure, renal failure, infectious diseases, and deep vein thrombosis [13]. Therefore, surgeons must pay attention to the management and prevention of postoperative anemia.

To further improve postoperative QOL and patient satisfaction, the importance of preventing postoperative anemia after THA has become increasingly important. Hence, our study developed a nomogram via R language to assess the probability of postoperative anemia in patients. In recent years, nomograms have been extensively applied in clinical medical predictions, such as in survival probability analysis or adverse event prediction. This graphical representation of regression analysis outcomes could assist medical personnel in assessing whether THA patients are at risk for postoperative anemia, and facilitate the implementation of appropriate interventions. Our study expands on previous prediction models by shifting our endpoint from transfusion to anemia, setting it apart from past studies [5, 14, 15].

Our findings indicate that factors such as low BMI, low preoperative Hb levels, long surgery duration, high intraoperative blood loss, high preoperative SAA levels, history of cerebrovascular disease, and history of osteoporosis all increase the probability of postoperative anemia in patients undergoing primary THA surgery.

Being underweight is often viewed as a sign of chronic disease in patients [16]. Low BMI, to a certain extent, reflects the nutritional status of the body. Older patients, who may be frail or malnourished, struggle to maintain normal body reserves. Due to this diminished reserve capacity, acute stress injuries such as surgery can readily result in postoperative anemia in these patients. Furthermore, patients with low BMI usually have relatively low body weight and blood volume. Anoushiravani et al. reported that such patients face a higher risk of postoperative anemia and cardiac complications after THA [17]. Another study by Arshi et al. determined that low BMI is an independent risk factor for postoperative transfusion [18]. These are similar to our results.

Currently, there are limited reports available on the impact of osteoporosis on perioperative anemia in THA. In this study, we discovered that patients with osteoporosis experience a higher risk of postoperative anemia following THA. As age increases, bone quality progressively decreases. Patients with osteoporosis exhibit decreased bone density and thinned or even fractured trabeculae, which widens the bone marrow space. Blood can enter this space following proximal femoral osteotomy, which leads to blood loss. Additionally, osteoporotic patients have higher levels of oxygen free radicals in their bodies, which might interact with the enterocyte phospholipid membrane, leading to hemolysis and further hemoglobin loss [19].

Many studies have confirmed the correlation between preoperative Hb levels and the risk of postoperative blood transfusion in THA. Starlinger et al.'s study classified preoperative Hb levels as an independent risk factor for predicting postoperative transfusion [20]. The lower the patient's preoperative RBC, Hb, and HCT, the worse their ability to compensate for intraoperative bleeding, and the higher their risk of perioperative anemia. A retrospective study of 2,467 THA patients conducted by Choi et al. reported that lower preoperative Hb levels increase the risk of postoperative anemia [21]. Our study supports these findings, showing that preoperative Hb level is an independent risk factor for postoperative anemia following THA. Our study further notes that for every decrease of 20 g/L in preoperative Hb levels, the nomogram model score is increased by 15 weighted points.

This investigation identified intraoperative blood loss and operation time as risk factors for postoperative anemia following THA, which is consistent with previous research findings [5, 22]. A prolonged operation time often indicates a more complicated procedure with greater damage, leading to increased bleeding. Meanwhile, an extended operation time can trigger a more severe inflammatory response, which further exacerbates hemolysis. According to related studies, for every 15-min increase in surgical time, the risk of transfusion was found to increase by 9% [23]. Intraoperative blood loss serves as the most direct indicator for calculating Hb decrease and is a primary cause of anemia. Hidden blood loss due to intraoperative blood permeating into tissues, residual blood in the medullary cavity, insufficient hemostasis, and hemolysis contributes to increased blood loss. Therefore, the actual total blood loss (TBL) during THA typically exceeds the observed intraoperative blood loss [24, 25]. Additionally, patients with extensive intraoperative blood loss and prolonged operation time typically experience various complications, further impeding their postoperative recovery.

Another surprising finding were the risks associated with preoperative SAA concentration and history of cerebrovascular disease, which are factors rarely mentioned. SAA, a nonspecific acute-phase reactive protein, is mainly produced by liver cells in response to cytokines interleukin (IL)-1β, IL-6, and tumor necrosis factor (TNF)-α. In normal people, the concentration of SAA is very low; however, it increases during instances of inflammation, such as in diabetes, stroke, and cerebral ischemia [26, 27]. Relevant studies have shown that SAA is a predictor of diabetes [28]. Regarding the history of cerebrovascular disease, our findings contrast with Zhu et al., who found no connection between a history of cerebrovascular disease and postoperative blood transfusion [29, 30]. We speculate this discrepancy may be linked to the long-term use of anticoagulants by patients with cerebrovascular diseases, and further research is still needed.

Our nomogram significantly increases the predictive and diagnostic abilities for postoperative anemia post-THA and forms the basis for devising preoperative treatment strategies to mitigate the risk of postoperative anemia in high-risk patients. For low-risk cases, the corresponding interventions can be reduced to ease the patient's financial burden.

This study has several limitations. First, the relatively small sample size may have introduced bias. Therefore, future studies should aim to validate these findings through increased patient recruitment or multicenter research. Secondly, the retrospective nature of this study limits the level of evidence. Thirdly, the study primarily centered on the impact of preoperative risk factors on postoperative anemia, without the long-term follow-up of patient complications. To address these limitations, subsequent research will be conducted. Future investigations will need to incorporate more cases, evaluate the accuracy of postoperative blood transfusion predictions with modeling, expand the range of the studied sample to include other local and regional centers, and conduct external validation to determine the practical value of the model.

## Conclusions

This study created a nomogram model based on BMI, operation time, intraoperative bleeding, Hb, SAA, history of cerebrovascular disease, and history of osteoporosis value as independent risk factors for postoperative anemia, with good indexing and accuracy that can provide scientific guidance for individualized clinical prevention postoperative anemia with THA surgery.

## Data Availability

The datasets used and/or analyzed during the current study are available from the corresponding author on reasonable request.

## Abbreviations

ASA: American Society of Anesthesiologists
DCA: Decision curve analysis
QOL: Quality of life
ROC: Receiver operating characteristic
TBL: Total blood loss
THA: Total hip arthroplasty
TXA: Tranexamic acid
BMI: Body mass index
CRP: C-reactive protein
CSEA: Combined spinal epidural anesthesia
GA: General anesthesia
GLB: Globulin
HCT: Hematocrit
MCH: Mean corpuscular hemoglobin
MCHC: Mean corpuscular hemoglobin concentration
MCV: Mean corpuscular volume
OAH: Osteoarthritis of hip
ONFN: Osteonecrosis of the femoral head
RBC: Red blood cell
SAA: Serum amyloid-a
TIBC: Total iron binding capacity
TP: Total protein
